# Whole Genome Sequence of the Probiotic Strain *Lactobacillus paracasei* N1115, Isolated from Traditional Chinese Fermented Milk

**DOI:** 10.1128/genomeA.00059-14

**Published:** 2014-03-13

**Authors:** Shijie Wang, Hong Zhu, Fang He, Yongkang Luo, Zhiyuan Kang, Chun Lu, Lili Feng, Xiaoli Lu, Yuling Xue, Hua Wang

**Affiliations:** a College of Food Science and Nutritional Engineering, China Agricultural University, Beijing, China; b Shijiazhuang Junlebao Dairy Co. Ltd., Shijiazhuang, Hebei, China; c Department of Nutrition and Food Safety, West China School of Public Health, Sichuan University, Chengdu, China

## Abstract

*Lactobacillus paracasei* N1115 is a new strain with probiotic properties isolated from traditional homemade dairy products in Inner Mongolia, China. Here, we report the complete genome sequence of *L. paracasei* N1115, which shows high similarity to the well-studied probiotic *Lactobacillus rhamnosus* GG, and 3 structures turned out to be inversions, according to the colinearity analysis of the BLAST alignment.

## GENOME ANNOUNCEMENT

*Lactobacillus paracasei* N1115 has been isolated from traditional dairy products in Inner Mongolia, China. A patent has been applied for this strain to be a probiotic since it exhibits high-level resistance to acid and bile stresses and it stimulates macrophages to produce interleukin 10 (IL-10), IL-6, and tumor necrosis factor alpha (TNF-α) (1). The whole genome of *L. paracasei* N1115 was sequenced by the Illumina HiSeq 2000 platform (performed by the Beijing Genomics Institute [BGI], Shenzhen, China).

High-molecular-weight genomic DNA was used to construct three genomic libraries, with 0.5-kb (914 Mb), 2-kb (250 Mb), and 6-kb (250 Mb) insert sizes, respectively. Using the Illumina HiSeq 2000 system, 1.4 Gbp clean data were given, which provided 420-fold genome coverage. Using SOAP*denovo* (http://soap.genomics.org.cn/soapdenovo.html) (2), the reads were assembled into 38 scaffolds and 268 contigs. Using the SOAPaligner (http://soap.genomics.org.cn/soapaligner.html) tool, 96.2% of the reads were mapped to assembly sequences. Most of the gaps within the scaffolds were ﬁlled by local assembly and overlap extension with paired-end reads. We also filled the gaps between scaffolds by sequencing PCR products using an ABI 3730 capillary sequencer.

The complete genome of N1115 contains a single circular chromosome of 2,938,059 bp (46.59% G+C) and four plasmids (pN1 [3,833 bp], pN2 [55,121 bp], pN3 [58,511 bp], and pN4 [8,755 bp]). The overall G+C content is 46.45%, whereas the four plasmids have G+C contents of 43.26% on average. This genome size is slightly larger than the complete genomes of *Lactobacillus rhamnosus* ATCC 53103 (3.010 Mb) and *Lactobacillus casei* ATCC 334 (2.925 Mb). The complete genome of N1115 contains 3,063 predicted gene sequences with 2,584,095 bp (2–6), covering 84.33% of the genome. N1115 also harbors 15 rRNA operons and 61 tRNAs (7, 8).

A comparative genome analysis was made between the assembly sequence and reference sequence, and 3 structural variations were found. Based on the colinearity analysis of the BLAST alignment, 3 structural variations turned out to be inversions (an inversion is a chromosome rearrangement in which a segment of a chromosome is reversed end to end). All 3 inversions were verified by PCR amplification.

A significant comparative genome analysis showed that N1115 carries most of the core genes of *L. casei*, and no known pathogenic genes were identiﬁed. A comparative genomic analysis was performed with the published genomes of *L. casei* ATCC 334 and *L. rhamnosus* GG. In conclusion, the genome sequence provides new avenues to further explore the gene-based functional and probiotic mechanisms of N1115. In addition, comparative and functional genomics analyses might also be carried out to trace the origin and evolution of this bacterium.

### Nucleotide sequence accession numbers.

The *L. paracasei* N1115 chromosome and plasmid pN1, pN2, pN3, and pN4 sequences have been deposited in GenBank under accession no. CP007122, CP007123, CP007124, CP007125, and CP007126, respectively.

## References

[1] Zhu H Wang S Lu C Feng L Lu X Kang Z Cui S 2011 *Lactobacillus casei* N1115, its immunoregulation effect and application. Chinese patent no. ZL 201110357058.8

[2] Li R Zhu H Ruan J Qian W Fang X Shi Z Li Y Li S Shan G Kristiansen K Li S Yang H Wang J Wang J 2010 *De novo* assembly of human genomes with massively parallel short read sequencing. Genome Res. 20:265–272. 10.1101/gr.097261.109 20019144 PMC2813482

[3] Delcher AL Bratke KA Powers EC Salzberg SL 2007 Identifying bacterial genes and endosymbiont DNA with Glimmer. Bioinformatics 23:673–679. 10.1093/bioinformatics/btm009 17237039 PMC2387122

[4] Bairoch A Apweiler R 2000 The Swiss-Prot protein sequence database and its supplement TrEMBL in 2000. Nucleic Acids Res. 28:45–48. 10.1093/nar/28.1.45 10592178 PMC102476

[5] Zdobnov EM Apweiler R 2001 InterProScan—an integration platform for the signature-recognition methods in InterPro. Bioinformatics 17:847–848. 10.1093/bioinformatics/17.9.847 11590104

[6] Ashburner M Ball CA Blake JA Botstein D Butler H Cherry JM Davis AP Dolinski K Dwight SS Eppig JT Harris MA Hill DP Issel-Tarver L Kasarskis A Lewis S Matese JC Richardson JE Ringwald M Rubin GM Sherlock G 2000 Gene Ontology: tool for the unification of biology. The Gene Ontology Consortium. Nat. Genet. 25:25–29. 10.1038/75556 10802651 PMC3037419

[7] Kanehisa M Goto S 2000 KEGG: Kyoto encyclopedia of genes and genomes. Nucleic Acids Res. 28:27–30. 10.1093/nar/28.1.27 10592173 PMC102409

[8] Griffiths-Jones S Moxon S Marshall M Khanna A Eddy SR Bateman A 2005 Rfam: annotating non-coding RNAs in complete genomes. Nucleic Acids Res. 33:D121–D124. 10.1093/nar/gki081 15608160 PMC540035

